# Correlation of growth differentiation factor 15 level in esophageal cancer with cachectic indicators and postoperative infectious complication

**DOI:** 10.1007/s10388-025-01157-0

**Published:** 2025-09-29

**Authors:** Yohei Ozawa, Hiroshi Okamoto, Yusuke Taniyama, Chiaki Sato, Hirotaka Ishida, Takeya Sato, Shinobu Ohnuma, Michiaki Unno, Takaaki Abe, Takashi Kamei

**Affiliations:** 1 Department of Surgery, Tohoku University Graduate School of Medicine, 1-1 Seiryo-Machi, Aoba-ku, Sendai, Miyagi Japan; 2 Department of Clinical Biology and Hormonal Regulation, Tohoku University Graduate School of Medicine, Sendai, Japan

**Keywords:** Esophageal cancer, GDF-15, Sarcopenia, Cachexia, Malnutrition

## Abstract

**Background:**

Patients with esophageal cancer (EC) usually have multiple comorbidities, particularly, high cachexia incidence, which may lead to increased postoperative complications. A novel inflammatory marker, growth differentiation factor 15 (GDF15), was recently reported to be associated with cancer cachexia. This study evaluated the correlation between clinical data suggestive of cachexia in patients with EC and circulating GDF15 levels.

**Methods:**

Eighty patients with EC were enrolled in this study. Plasma samples were collected before initiating any cancer treatment. GDF15 was quantified using ELISA. Clinical information, including age, comorbidities, biochemical data, Controlling Nutritional Status score, and Psoas muscle index (PMI), was collected from the clinical records. Clinical impact of GDF15 was then evaluated and compared with cachectic indicators or postoperative results.

**Results:**

The median value of GDF15 was 1168 pg/mL (range 298–9100 pg/mL). GDF15 values statistically correlated with age, prevalence of diabetes, serum level of aspartate aminotransferase/γ-glutamyltransferase/creatinine/blood sugar/albumin, and PMI. Sixty-three patients finally underwent curative esophagectomy with right thoracic approach and gastric tube reconstruction. Patients with infectious complications had a statistically higher GDF15 than those without. The cut-off value was 930 pg/mL for detecting infectious complications, with an area under the receiver operating characteristic curve value of 0.685, and high GDF15 was detected as an independent risk factor for postoperative infectious complications.

**Conclusions:**

GDF15 is potentially suggestive of general condition deterioration from aging, organ dysfunction, and decreased muscle mass, which may lead to cachexia in patients with EC. Moreover, patients with higher GDF15 are at a risk of postoperative infectious complications.

**Supplementary Information:**

The online version contains supplementary material available at 10.1007/s10388-025-01157-0.

## Introduction

Cachexia was first defined as “a complex metabolic syndrome associated with underlying illness and characterized by loss of muscle with or without loss of fat mass” by Evans et al. [1]. Cachexia is seen in patients with various underlying diseases and is known to be an important prognostic indicator in patients with cancer. However, the condition of patients with cachexia is difficult to qualify because it is formed by a complex interaction of various factors. Although there are several well-known indicators that can estimate the cachectic condition, including several symptoms such as loss of appetite, loss of body weight, decreased body mass index, decreased grip strength, and increased C-reactive protein [2–4], there are only few single blood markers that can reflect cancer cachexia caused by complex triggers.

Growth differentiation factor 15 (GDF15) also known as macrophage inhibitory cytokine-1 (MIC-1) is in a member of the transforming growth factor beta super family. DGF15 is weakly expressed in the most organs including liver, lung, and kidneys under normal conditions, but its expression is known to be elevated in inflammatory condition such as organ failure or cancer bearing patients. Therefore, GDF15 was recently reported to be associated with cancer cachexia. In basic research, circulating GDF15 levels have been correlated with the loss of food intake, tumor size, and skeletal muscle atrophy in tumor-bearing animals [5–7]. In the clinical field, several investigators have reported that circulating GDF15 levels in patients with cachectic cancer are much higher than those in non-cachectic patients, and this was correlated with cancer-associated weight loss [7, 8]. These previous studies suggest that GDF15 is potentially useful as a clinical cachectic biomarker for patients with cancer, particularly those facing highly invasive surgery. However, there are a few reports on the impact on perioperative outcomes. Therefore, in this study, we evaluated the correlation between clinical data suggesting cachexia in patients with Esophageal Cancer (EC) and plasma GDF15 to assess the usefulness of GDF15 as a potential biomarker for cachexia and risk factors for postoperative complications.

## Methods

### Patient selection and information

**Fig. 1:**
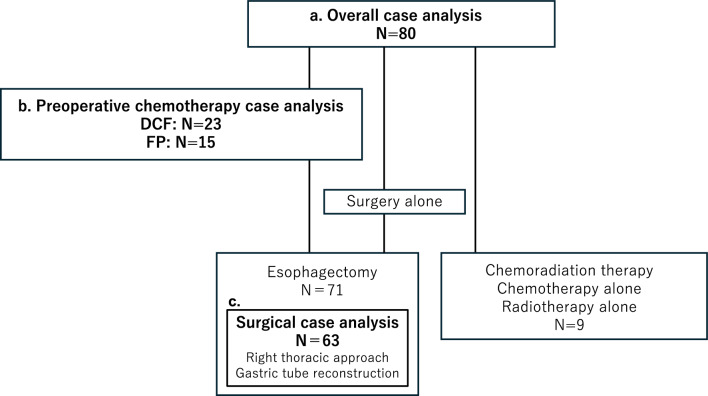
Selected treatment and analysis cohort in this study. **a** The patients of overall case analysis were used for comparison between GDF15 level and patient’s characteristics, nutritional status, blood test, and tumor progression. **b** The patients of preoperative chemotherapy case analysis were used for comparison between GDF15 level and chemotherapeutic adverse events and its response. **c** The patients of surgical case analysis were used for comparison between GDF15 level and surgical complications

Eighty patients with EC who first visited our outpatient ward between July 2021 and March 2023 were enrolled in this study. After careful additional examination, the treatment was determined based on EC practice guidelines 2022 edited by the Japanese esophageal society [9, 10]. Patient information, including age, sex, comorbidities, social history, and blood test results at the time of pre-treatment, were collected from clinical records. Alcohol consumption was defined only for patients who habitually consumed alcohol. Chronic obstructive pulmonary disease was defined as the patients < 70% of FEV1.0%. The tumor stage was diagnosed according to the TNM classification of malignant tumors, 8th edition [11]. In case patients who underwent surgery, postoperative complications according to the Clavien–Dindo classification of surgical complications and results of neoadjuvant chemotherapy (NAC) were also collected. Adverse event for NAC was determined by Common Terminology Criteria for Adverse Events version 5.0. The response to NAC was determined using the Japanese classification of esophageal cancer [12]. We created three different cohorts for each purpose of our analysis, namely, an "Overall case analysis" to examine the background and GDF15 levels of esophageal cancer patients, a “Preoperative chemotherapy case analysis” to examine adverse events of NAC, and a “Surgical case analysis” to examine surgical complications (Fig. 1). The patients with “Surgical case analysis” were limited to right thoracic approach and gastric tube reconstruction. All patients consented to pre-treatment blood collection for research purposes and the study protocol prior to blood collection. The study protocol was approved by the Ethics Committee of the Tohoku University Graduate School of Medicine (accession no. 2021-1-167).

### Collection of plasma samples

Plasma samples were collected from all patients at the time of their first visit or admission. All samples were obtained prior to any treatment for EC and these were stored at 4 °C before dispensing 200 μL each and then stored at − 80 °C until further analysis.

### ELISA for GDF-15

Plasma GDF15 concentrations were quantified using a Human GDF-15 Quantikine ELISA Kit (R&D Systems, Inc., Minneapolis, MN, USA). After defrosting samples on ice, each 13 µL of samples and 50 µL of standards adjusted concentration were put into each well which pre-aliquoted assay diluent and incubated at room temperature for 2 h. Subsequently, after aspiration and washing each well, 200 µL conjugate was put into each well and incubated at room temperature for 1 h. Finally, each standard and sample were reacted with the substrate and stop solutions, and the absorbance was read using a spectrometer with a wavelength correction set to 540 nm. Detailed measurement methods were obtained from the manufacturer’s instructions and previous reports [13, 14].

### Parameters for patient’s malnutrition and sarcopenia

To evaluate the correlation between GDF-15 levels and malnutrition and sarcopenia, we chose the controlling nutritional status (CONUT) score and psoas muscle index (PMI) as the parameters of malnutrition and sarcopenia, respectively. CONUT score was calculated by using serum total cholesterol, albumin, and total lymphocyte at the time of pre-treatment and malnutrition was defined as CONUT score ≥ 2. Sarcopenia was measured using the cross-sectional areas of both psoas muscles at the caudal part of the third lumbar vertebra on pre-treatment computed tomography images. PMI was calculated as follows:

$${\mathrm{PMI}} = {\mathrm{cross}} {\mathrm{-}} {\text{sectional area of both psoas muscles }}({\mathrm{cm}}^{{2}} )/{\mathrm{height}}^{{2}}\; ({\mathrm{m}}^{{2}} ).$$

Sarcopenia was defined as that lower than the sex-specific 25th percentile [15].

### Statistical analysis

Mann–Whitney *U* test was used to analyze the relationship between GDF15 levels, patient characteristics, and postoperative complications. Linear regression analysis was performed to estimate the relationship between patients’ age or blood data and GDF15 levels. Multivariate analysis was performed using logistic regression, and the variables used for the multivariate analyses were determined using stepwise regression. Receiver operating characteristic (ROC) curves and the area under the curve (AUC) were calculated to determine the optimal cut-off value for predicting postoperative infectious complications. Statistical significance was set at *P* < 0.05. All statistical analyses were performed using JMP Pro 16 software (SAS Institute, Cary, NC, USA).

## Results

### Patient’s characteristics

A total of 80 patients were enrolled in this study. The mean age was 68.3 years and 80.0% were male. The number of patients diagnosed with cStages I, II, III, and IV was 21, 10, 32, and 17, respectively. Histological subtypes were classified as squamous cell carcinoma, adenocarcinoma, and others (including basaloid squamous cell carcinoma, and carcinoma), with 61, 17, and 2, respectively. Surgery alone, NAC, and other treatments (including chemoradiotherapy, chemotherapy alone, and radiotherapy alone) were selected for 35, 38, and 7 patients, respectively. The number of patients with malnutrition diagnosed using CONUT was 24 (30.0%) and that of patients with sarcopenia was 21 (26.3%). The median value of GDF15 was 1168 pg/mL (range 298–9100 pg/mL). The patient characteristics are presented in Table 1.

**Table 1:** Patient’s characteristics

	Overall case analysis (*N* = 80)	%	Surgical case analysis (*N* = 63)	%
Age, years				
Mean ± SD	68.3 ± 8.8		68.5 ± 8.3	
Sex				
Male	64	80.0	49	77.8
Female	16	20.0	14	22.2
Performance status				
PS0	70	87.5	57	90.5
PS1	10	12.5	6	9.5
Smoking history	64	80.0	48	76.2
Alchohol consumption^a^	68	85.0	53	84.1
Comorbidities				
COPD^b^	20	25.0	15	23.8
Hypertension	54	67.5	44	69.8
Diabetes	12	15.0	7	11.1
Dysphagia^c^	42	52.5	33	52.4
cT stage				
T1–2	35	43.8	30	47.6
T3–4	45	56.3	33	52.4
cN stage				
N negative	27	33.8	24	38.1
N positive (N1, N2, N3)	53 (33, 20, 0)	66.3	39 (25, 14, 0)	61.9
cM stage				
M0	67	83.8	57	90.5
M1	13	16.3	6	9.5
cStage				
I–II	31 (21, 10)	38.8	28 (20, 8)	44.4
III–IV	49 (32, 17)	61.3	35 (27, 8)	55.6
Tumor location				
CeUtMt (Ce, Ut, Mt)	40 (1, 10, 29)	50.0	32 (1, 8, 23)	50.8
LtAeG (Lt, Ae, G)	40 (26, 13, 1)	50.0	31 (22, 9, 0)	49.2
Histological subtype				
Squamous cell carcinoma	61	76.3	49	77.8
Adenocarcinoma	17	21.3	13	20.6
Other	2	2.5	1	1.6
Initial treatment				
Surgery	35	43.8	28	44.4
Neoadjuvant chemotherapy (FP, DCF)	38 (15, 23)	47.5	34	54.0
Other	7	8.8	1	1.6
Neoadjuvant chemotherapy				
FP	15	18.5	15	44.1
DCF	23	28.4	19	55.9
BMI, kg/m^2^				
Mean ± SD	22.5 ± 3.0		22.6 ± 3.0	
CONUT				
0–1	56	70.0	42	66.7
2–12	24	30.0	21	33.3
Sarcopenia				
Sarcopenia	21	26.3	16	25.4
Non-sarcopenia	59	73.8	47	74.6
GDF-15, pg/mL				
Median (range)	1168 (298–9100)		1103 (413–9100)	

^a^Habtal alchohol consumption

^b^COPD defined as < 70% of FEV1.0%

^c^Dysphagia score ≥ 1

### GDF15 correlated with cachectic indicators but not with tumor progression

**Fig. 2:**
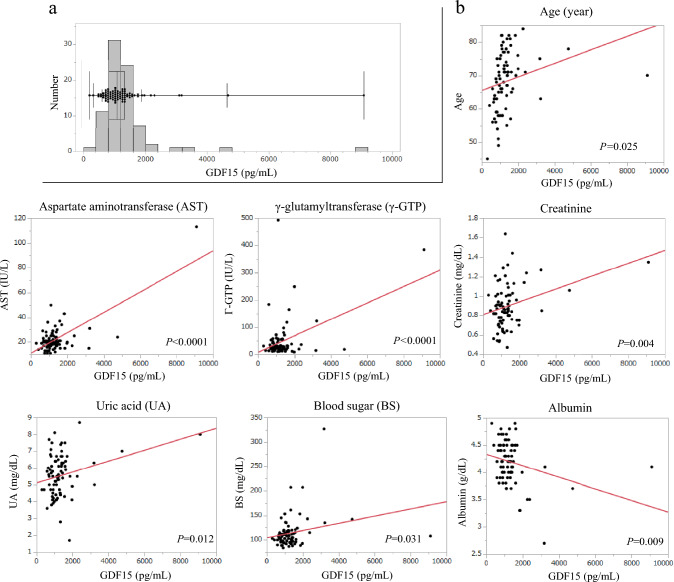
GDF15 distribution and correlation between continuous variables. **a** Plasma GDF15 distribution. **b** Correlation between age, blood biochemical data, and circulating growth differentiation factor 15 (GDF15). GDF15 escalations are seen well correlated with escalation of age, and serum levels of aspartate aminotransferase (AST), γ-glutamyltransferase (γ-GTP), creatinine, uric acid (UA), and blood sugar (BS)

Correlation between GDF15 and patient characteristics are shown in Table 2. Both categorical and continuous variables of age were strongly correlated with GDF15 (Fig. 2 and Table 2) (elder vs. younger: 1323 vs. 903, *P* < 0.001). Those with a worse tendency performance status (PS) (PS 0 vs. 1: 1116 vs. 1331, *P* = 0.052) were also observed. Patients with diabetes had significantly higher GDF15 than unaffected patients (1449 vs. 1117, *P* = 0.021). Furthermore, sarcopenia patients also had higher GDF15 than non-sarcopenia patients (1360 vs. 1076, *P* = 0.003). The malnutrition indicator CONUT score was not significantly correlated with GDF15, but a correlation between a worse CONUT score and GDF15 increase was observed. In each element of CONUT, namely, total lymphocyte, total cholesterol, and albumin, only serum albumin was observed negative correlation with GDF15 (*P* = 0.009) (Fig. 2). In correlation between GDF15 and the result of blood test, GDF15 was positively correlated with aspartate aminotransferase (*P* < 0.0001), γ-glutamyltransferase (*P* < 0.0001), creatinine (*P* = 0.004), uric acid (*P* = 0.012), and blood sugar (*P* = 0.031) (Fig. 2). All the results we examined are shown in Supplementary Fig. 1.

**Table 2:** Patient’s characteristics vs GDF15

	GDF15, pg/mL [median (range)]	*P*-value
Age (< 68/≥ 68)	903 (298–3220)/1323 (795–9100)	<** 0**.**001**
Sex (male/female)	1207 (298–9100)/1054 (583–1971)	0.361
Performance status (0/1)	1116 (298–9100)/1331 (989–2378)	0.052
Smoking history (yes/no)	1206 (298–9100)/978 (584–1971)	0.107
Alchohol consumption^a^ (yes/no)	1206 (414–9100)/1030 (298–1971)	0.241
Comorbidities		
COPD^b^ (yes/no)	1297 (768–2379)/1101 (298–9100)	0.128
Hypertension (yes/no)	1206 (414–9100)/994 (298–4743)	0.071
Diabetes (yes/no)	1449 (941–3220)/1117 (298–9100)	**0**.**021**
Dysphagia^c^ (yes/no)	1102 (584–4743)/1217 (298–9100)	0.603
cT stage (T1–2/T3–4)	1214 (298–9100)/1103 (582–4743)	0.631
cN stage (negative/positive)	1076 (414–9100)/1199 (298–4743)	0.448
cM stage (M0/M1)	1190 (298–9100)/1076 (642–3220)	0.710
cStage (I–II/III–IV)	1165 (298–9100)/1170 (582–4743)	0.763
Tumor location (CeUtMt/LtAeG)	1088 (414–9100)/1195 (298–3179)	0.693
Histological subtype (SCC/AC/other)	1214 (414–9100)/1027 (298–3179)/1226 (1063–1390)	0.254
BMI, kg/m^2^ (< 23/≥ 23)	1199 (298–9100)/1165 (414–3220)	0.437
CONUT (0–1/2–12)	1117 (298–3220)/1333 (583–9100)	0.156
Sarcopenia (Yes/No)	1360 (642–9100)/1076 (298–3220)	**0**.**003**

Overall case analysis

^a^Habtal alchohol consumption

^b^COPD defined as < 70% of FEV1.0%

^c^Dysphagia score ≥ 1

In contrast, GDF15 levels did not correlate with tumor location, histological subtype, or tumor progression.

### GDF15 had no statistical correlation with NAC adverse event and response

Thirty-eight patients received NAC (23 for the docetaxel, cisplatin, plus 5-FU and 15 for the cisplatin plus 5-FU regimen). GDF15 was not correlated with any adverse events, including leukopenia, neutropenia, increased creatinine, anorexia, nausea, or mucositis oral and pathological response (Table 3).

**Table 3:** NAC adverse event and response vs GDF15

	DCF (*N* = 23)		FP (*N* = 15)	
	GDF15 [median (range)]	*P*-value	GDF15 [median (range)]	*P*-value
Leukopenia (≥ G2) (yes/no)	1029 (584–1860)/1310 (830–1437)	0.710	1392 (1099–1830)/1082 (802–1482)	0.112
Neutropenia (≥ G3) (yes/no)	1029 (584–1860)/1355 (1355)	0.651	1099 (802–1830)/1102 (848–1482)	1.000
Creatinine increased (≥ G1) (yes/no)	1364 (704–1689)/1029 (584–1860)	0.268	1296 (1033–1482)/1099 (802–1830)	0.151
Anorexia (≥ G2) (yes/no)	1029 (830–1437)/1223 (584–1860)	0.951	1099 (848–1393)/1102 (802–1830)	0.903
Nausea (≥ G2) (yes/no)	1373 (902–1437)/986 (584–1860)	0.224	1099 (1099)/1102 (802–1830)	0.817
Mucositis oral (≥ G2) (yes/no)	902 (584–1354)/1147 (704–1860)	0.300	1011 (989–1033)/1103 (802–1830)	0.235
Overall adverse events^a^ (yes/no)	1223 (796–1617)/1029 (584–1860)	0.806	1246 (989–1830)/1101 (802–1482)	0.361
Pathological response (Grade 1a/Grade 1b–2)	902 (796–1360)/1355 (584–1860)	0.322	1033 (848–1830)/987 (802–1393)	0.361

Preoperative chemotherapy case analysis

^a^Four or more of G2, or 3 or more G3 adverse events

### GDF15 correlated with infectious complication

Seventy-one patients underwent curative esophagectomy. Of these, 35 underwent robot-assisted minimally invasive esophagectomy, 33 underwent conventional (thoracoscopic) minimally invasive esophagectomy, two underwent transhiatal esophagectomy, and one underwent thoracotomy. Sixty-three (88.7%) patients underwent reconstruction via gastric conduit, five via small intestine, and three via secondary reconstruction. No perioperative mortalities were observed. To avoid the influence of surgical factors on postoperative complications as much as possible, this analysis was limited to the patients who underwent esophagectomy with right thoracic approach and gastric tube reconstruction. Cardiovascular complications, pneumonia, and anastomotic leakage were observed in 4 (6.3%), 11 (17.5%), and 5 (7.9%) patients, respectively. Cardiovascular complications were included 3 patients with atrial fibrillation and 1 patient with heart failure due to cardiac tamponade. The patients who developed infectious complications (including pneumonia, anastomotic leakage, and surgical site infection) had statistically higher GDF15 than those who did not (*P* = 0.024) (Table 4).

**Table 4:** Postoperative complications vs GDF15

	Cases (%)	GDF15, pg/mL [median (range)]	*P*-value
Cardiovascular (≥ GII) (yes/no)	4 (6.3)	1174 (929–1390)/1103 (414–9100)	0.757
Peumonia (≥ GII) (yes/no)	11 (17.5)	1310 (801–9100)/1099 (414–4743)	0.107
Anastomotic leakage (≥ GII) (yes/no)	5 (7.9)	1359 (1165–4743)/1099 (414–9100)	0.060
Surgical site infection (≥ GII) (yes/no)	3 (4.8)	958 (830–1103)/1148 (414–9100)	0.349
Infectious complication^a^ (≥ GII) (yes/no)	18 (28.6)	1262 (801–9100)/1071 (414–1860)	**0**.**024**
Overall complications (≥ GII) (yes/no)	29 (46.0)	1182 (584–9100)/1099 (414–1860)	0.334
Overall complications (≥ GIIIa) (yes/no)	14 (22.2)	1318 (802–4743)/1099 (298–9100)	0.089
Overall complications (≥ GIIIb) (yes/no)	5 (7.9)	1310 (1063–1390)/1102 (414–9100)	0.360

Surgical case analysis

^a^Patients with pneumonia and/or anastomotic leakage and/or surgical site infection

### Cut-off value of GDF15 for predicting infectious complication

The area under the curve of GDF15 by receiver operating characteristic curve for predicting postoperative infectious complications was 0.685 (Supplementary Fig. 2). The cut-off value was determined to be 930 pg/mL, with 41.9% sensitivity and 88.9% specificity. Stepwise multivariate logistic regression analysis estimated that tumor location (*P* = 0.001) and plasma GDF15 concentration (*P* = 0.020) were risk factors for infectious complications (Table 5).

**Table 5:** Univariate and multivariate analysis for infectious complication

	Univariate analysis			Multivariate analysis		
	Odds ratio	95% CI	*P*-value	Odds ratio	95% CI	*P*-value
Age (≥ 68/< 68)	3.284	0.995–10.841	0.051			
Sex (female/male)	1.079	0.285–4.090	0.911			
Performance status (1/0)	5.857	0.966–35.524	0.055			
Smoking history (yes/no)	1.515	0.364–6.314	0.568			
COPD^a^ (no/yes)	1.203	0.326–4.438	0.781			
Hypertension (yes/no)	1.690	0.469–6.085	0.422			
Diabetes (no/yes)	1.053	0.185–6.002	0.954			
Dysphagia^b^ (yes/no)	3.284	0.995–10.841	0.051			
cT stage (T1–2/T3–4)	1.450	0.382–3.459	0.804			
cN stage (positive/negative)	2.058	0.624–6.794	0.236			
cM stage (M0/M1)	1.275	0.124–13.147	0.838			
cStage (III–IV/I–II)	1.087	0.360–3.285	0.883			
Tumor location (CeUtMt/LtAeG)	9.333	2.327–37.442	**0**.**002**	17.16	3.378–87.206	**0.001**
BMI, kg/m^2^ (< 23/≥ 23)	1.646	0.537–5.048	0.383			
CONUT (2–12/0–1)	1.644	0.516–5.236	0.400			
Sarcopenia (yes/no)	3.500	1.048–11.690	**0**.**042**	3.57	0.730–17.557	0.116
GDF15 (≥ 930 pg/mL/< 930)	5.760	1.175–28.244	**0**.**031**	8.556	1.409–51.954	**0**.**020**

Surgical case analysis

^a^COPD defined as < 70% of FEV1.0%

^b^Dysphagia score ≥ 1

## Discussion

In this study, we assessed the usefulness of circulating GDF15 as a potential biomarker of cachexia and a risk factor for postoperative complications in patients with EC. Our results showed that circulating GDF15 levels were significantly higher in various indicators suggesting cachexia, such as advanced age, diabetes, and skeletal muscle loss which are important factors for sarcopenia. These results indicate that the elevated GDF15 may not be caused primarily by cancer, but by chronic inflammation in multiple organs caused by the patient’s comorbidities, life history, and aging.

DGF15 is normally synthesized in the liver and is thought to be expressed in the lungs and kidneys. However, its expression is known to be elevated in cells of inflamed tissues, such as in chronic renal failure [14, 16]. Many patients with EC have an older onset, and a history of heavy smoking and alcohol consumption which may predispose them to chronic inflammation in multiple organs where GDF15 can potentially be elevated. Moreover, in the current study, blood levels of parameters such as liver function, renal function, and blood sugar were correlated with GDF15. In contrast, oncological factors such as TNM staging, tumor location, and histological subtype were not correlated with GDF15. Although several previous studies have reported that patients with advanced cancer have higher levels of circulating GDF15 which is different from the results of the current study [8], these reports mainly focused on patients with unresectable advanced cancer, which may have caused the difference in results.

In the past decade, various perioperative indicators have been reported to influence postoperative complications of curative esophagectomy. Especially, sarcopenic and malnutrition status are well-known risk factor for infectious complication as well as worse prognosis [17–23]. However, no useful single blood markers have been reported as indicators of cachexia, malnutrition, or sarcopenia. Since our current data have shown that circulating GDF15 is a risk factor for infectious complications in patients with EC, GDF15 may be useful for predicting blood biomarkers of cachexia which can lead to infectious complications in patients with EC who have undergone curative esophagectomy.

Regarding GDF15 function, Hsu et al. have reported GDF15 regulates food intake by connecting brainstem-restricted receptor named grail cell-derived neurotrophic factor receptor alpha like [24]. Additionally, in cancer field, several basic and clinical reports have shown GDF15 inducing anorexia and following weight loss [6, 8]. These reports indicate that GDF15 escalation induces anorexia and that controlling GDF15 may suppress anorexia. In other words, not only active intervention, such as nutritional therapy, rehabilitation, and diabetes control normalize GDF15, but also direct GDF15 suppression may also improve cancer inducing anorexia, one of the causes of cachexia, and decrease postoperative complications. As a first step in this validation, we are now beginning to examine pre- and postoperative GDF15 levels and mid- to long-term prognosis. In addition, GDF15 is currently receiving much attention as a biomarker for mitochondrial dysfunction [25]. The possibility that increasing GDF15 in cancer patients may reflect not only anorexia and chronic inflammation but also mitochondrial dysfunction which we currently under investigation.

In contrast, although we expected circulating GDF15 may influence NAC adverse events before this study was conducted, and there was no correlation between GDF15 and NAC adverse events, including anorexia. Because two different NAC regimens were included in this study, the number of patients in the cohort was very small, which may be one of the reasons. Therefore, re-verification is needed. This study had several limitations. First, this study was conducted retrospectively at a single institute for a single cancer type, although several histological subtypes were included. Second, only a small number of patients were included. Therefore, prospective studies targeting a larger number of patients are necessary.

In conclusion, this study suggested circulating GDF15 is potentially suggestive of deterioration of the general condition, resulting from aging, organ dysfunction, and decreased muscle mass, which may lead to cachexia in EC patients. Moreover, higher circulating GDF15 levels are associated with a risk of postoperative infectious complications.

## Supplementary Information

Below is the link to the electronic supplementary material.

Supplementary file1 **Supplementary Fig. 1** Correlation between blood biochemical data and circulating growth differentiation factor 15 (GDF15). (PPTX 101 KB)

Supplementary file2 **Supplementary Fig. 2** Receiver operating characteristic curve for predicting postoperative infectious complication by circulating GDF15. The area under the curve (AUC) of GDF15 is 0.678. The cut-off value is determined to be 940 pg/mL with 42.3% sensitivity and 87.0% specificity. (PPTX 40 KB)

## Data Availability

The data that support the findings of this study are not openly available due to reasons of sensitivity and are available from the corresponding author upon reasonable request.

## References

[1] Evans WJ, Morley JE, Argilés J, Bales C, Baracos V, Guttridge D, et al. Cachexia: a new definition. Clin Nutr. 2008;27:793–9. 10.1016/j.clnu.2008.06.013. 18718696 10.1016/j.clnu.2008.06.013

[2] Namikawa T, Marui A, Yokota K, Fujieda Y, Munekage M, Uemura S, et al. Frequency and prognostic impact of cachexia during drug treatment for unresectable advanced gastric cancer patients. Surg Today. 2022;52:1560–7. 10.1007/s00595-022-02493-9. 35322296 10.1007/s00595-022-02493-9

[3] Martin L, Muscaritoli M, Bourdel-Marchasson I, Kubrak C, Laird B, Gagnon B, et al. Diagnostic criteria for cancer cachexia: reduced food intake and inflammation predict weight loss and survival in an international, multi-cohort analysis. J Cachexia Sarcopenia Muscle. 2021;12:1189–202. 10.1002/jcsm.12756. 34448539 10.1002/jcsm.12756 PMC8517347

[4] Mallard J, Gagez AL, Baudinet C, Herbinet A, Maury J, Bernard PL, et al. C-reactive protein level: a key predictive marker of cachexia in lymphoma and myeloma patients. J Hematol. 2019;8:55–9. 10.14740/jh536. 32300444 10.14740/jh536 PMC7153683

[5] Luan Y, Zhang Y, Yu SY, You M, Xu PC, Chung S, et al. Development of ovarian tumour causes significant loss of muscle and adipose tissue: a novel mouse model for cancer cachexia study. J Cachexia Sarcopenia Muscle. 2022;13:1289–301. 10.1002/jcsm.12864. 35044098 10.1002/jcsm.12864 PMC8977964

[6] Borner T, Arnold M, Ruud J, Breit SN, Langhans W, Lutz TA, et al. Anorexia-cachexia syndrome in hepatoma tumour-bearing rats requires the area postrema but not vagal afferents and is paralleled by increased MIC-1/GDF15. J Cachexia Sarcopenia Muscle. 2017;8:417–27. 10.1002/jcsm.12169. 28025863 10.1002/jcsm.12169 PMC5476861

[7] Lerner L, Tao J, Liu Q, Nicoletti R, Feng B, Krieger B, et al. MAP3K11/GDF15 axis is a critical driver of cancer cachexia. J Cachexia Sarcopenia Muscle. 2016;7:467–82. 10.1002/jcsm.12077. 27239403 10.1002/jcsm.12077 PMC4863827

[8] Lerner L, Hayes TG, Tao N, Krieger B, Feng B, Wu Z, et al. Plasma growth differentiation factor 15 is associated with weight loss and mortality in cancer patients. J Cachexia Sarcopenia Muscle. 2015;6:317–24. 10.1002/jcsm.12033. 26672741 10.1002/jcsm.12033 PMC4670740

[9] Kitagawa Y, Ishihara R, Ishikawa H, Ito Y, Oyama T, Oyama T, et al. Esophageal cancer practice guidelines 2022 edited by the Japan Esophageal Society: part 2. Esophagus. 2023;20:373–89. 10.1007/s10388-023-00994-1. 36995449 10.1007/s10388-023-00994-1 PMC10235142

[10] Kitagawa Y, Ishihara R, Ishikawa H, Ito Y, Oyama T, Oyama T, et al. Esophageal cancer practice guidelines 2022 edited by the Japan Esophageal Society: part 1. Esophagus. 2023;20:343–72. 10.1007/s10388-023-00993-2. 36933136 10.1007/s10388-023-00993-2 PMC10024303

[11] Brierley JD, Gospodarowicz MK, Wittekind C, eds. TNM classification of malignant tumours. 8th ed. Wiley-Blackwell; 2017.

[12] Doki Y, Tanaka K, Kawachi H, Shirakawa Y, Kitagawa Y, Toh Y, et al. Japanese classification of esophageal cancer, 12th edition: part II. Esophagus. 2024;21:216–69. 38512393 10.1007/s10388-024-01048-w PMC11199314

[13] Oikawa Y, Izumi R, Koide M, Hagiwara Y, Kanzaki M, Suzuki N, et al. Mitochondrial dysfunction underlying sporadic inclusion body myositis is ameliorated by the mitochondrial homing drug MA-5. PLoS ONE. 2020;15:e0231064. 10.1371/journal.pone.0231064. 33264289 10.1371/journal.pone.0231064 PMC7710105

[14] Oshita T, Watanabe S, Toyohara T, Kujirai R, Kikuchi K, Suzuki T, et al. Urinary growth differentiation factor 15 predicts renal function decline in diabetic kidney disease. Sci Rep. 2023;13:12508. 10.1038/s41598-023-39657-7. 37532799 10.1038/s41598-023-39657-7 PMC10397309

[15] Ozawa Y, Nakano T, Taniyama Y, Sakurai T, Onodera Y, Kamiya K, et al. Evaluation of the impact of psoas muscle index, a parameter of sarcopenia, in patients with esophageal squamous cell carcinoma receiving neoadjuvant therapy. Esophagus. 2019;16:345–51. 10.1007/s10388-019-00670-3. 30980203 10.1007/s10388-019-00670-3

[16] Breit SN, Johnen H, Cook AD, Tsai VWW, Mohammad MG, Kuffner T, et al. The TGF-β superfamily cytokine, MIC-1/GDF15: a pleotrophic cytokine with roles in inflammation, cancer and metabolism. Growth Factors. 2011;29:187–95. 10.3109/08977194.2011.607137. 21831009 10.3109/08977194.2011.607137

[17] Makiura D, Ono R, Inoue J, Fukuta A, Kashiwa M, Miura Y, et al. Impact of sarcopenia on unplanned readmission and survival after esophagectomy in patients with esophageal cancer. Ann Surg Oncol. 2018;25:456–64. 10.1245/s10434-017-6294-4. 29214454 10.1245/s10434-017-6294-4

[18] Paireder M, Asari R, Kristo I, Rieder E, Tamandl D, Ba-Ssalamah A, et al. Impact of sarcopenia on outcome in patients with esophageal resection following neoadjuvant chemotherapy for esophageal cancer. Eur J Surg Oncol. 2017;43:478–84. 10.1016/j.ejso.2016.11.015. 28024944 10.1016/j.ejso.2016.11.015

[19] Elliott JA, Doyle SL, Murphy CF, King S, Guinan EM, Beddy P, et al. Sarcopenia: prevalence, and impact on operative and oncologic outcomes in the multimodal management of locally advanced esophageal cancer. Ann Surg. 2017;266:822–30. 10.1097/SLA.0000000000002398. 28796017 10.1097/SLA.0000000000002398

[20] Nishigori T, Okabe H, Tanaka E, Tsunoda S, Hisamori S, Sakai Y. Sarcopenia as a predictor of pulmonary complications after esophagectomy for thoracic esophageal cancer. J Surg Oncol. 2016;113:678–84. 10.1002/jso.24214. 26936808 10.1002/jso.24214

[21] Makiura D, Ono R, Inoue J, Kashiwa M, Oshikiri T, Nakamura T, et al. Preoperative sarcopenia is a predictor of postoperative pulmonary complications in esophageal cancer following esophagectomy: a retrospective cohort study. J Geriatr Oncol. 2016;7:430–6. 10.1016/j.jgo.2016.07.003. 27452909 10.1016/j.jgo.2016.07.003

[22] Harada K, Ida S, Baba Y, Ishimoto T, Kosumi K, Tokunaga R, et al. Prognostic and clinical impact of sarcopenia in esophageal squamous cell carcinoma. Dis Esophagus. 2016;29:627–33. 10.1111/dote.12381. 26123787 10.1111/dote.12381

[23] Hikage M, Taniyama Y, Sakurai T, Sato C, Takaya K, Okamoto H, et al. The influence of the perioperative nutritional status on the survival outcomes for esophageal cancer patients with neoadjuvant chemotherapy. Ann Surg Oncol. 2019;26:4744–53. 10.1245/s10434-019-07742-9. 31440925 10.1245/s10434-019-07742-9

[24] Hsu JY, Crawley S, Chen M, Ayupova DA, Lindhout DA, Higbee J, et al. Non-homeostatic body weight regulation through a brainstem-restricted receptor for GDF15. Nature. 2017;550:255–9. 10.1038/nature24042. 28953886 10.1038/nature24042

[25] Yatsuga S, Fujita Y, Ishii A, Fukumoto Y, Arahata H, Kakuma T, et al. Growth differentiation factor 15 as a useful biomarker for mitochondrial disorders. Ann Neurol. 2015;78:814–23. 10.1002/ana.24506. 26463265 10.1002/ana.24506 PMC5057301

