# Supplementary material for: Correlation of growth differentiation factor 15 level in esophageal cancer with cachectic indicators and postoperative infectious complication
Source: Esophagus. 2025 Sep 29;23(1):230–8. doi: 10.1007/s10388-025-01157-0 (PMC12832575; doi:10.1007/s10388-025-01157-0)
Supplement: Supplementary file 1 — Supplementary file1 Supplementary Fig. 1 Correlation between blood biochemical data and circulating growth differentiation factor 15 (GDF15). (PPTX 101 KB) [file 10388_2025_1157_MOESM1_ESM.pptx]

## Slide 1
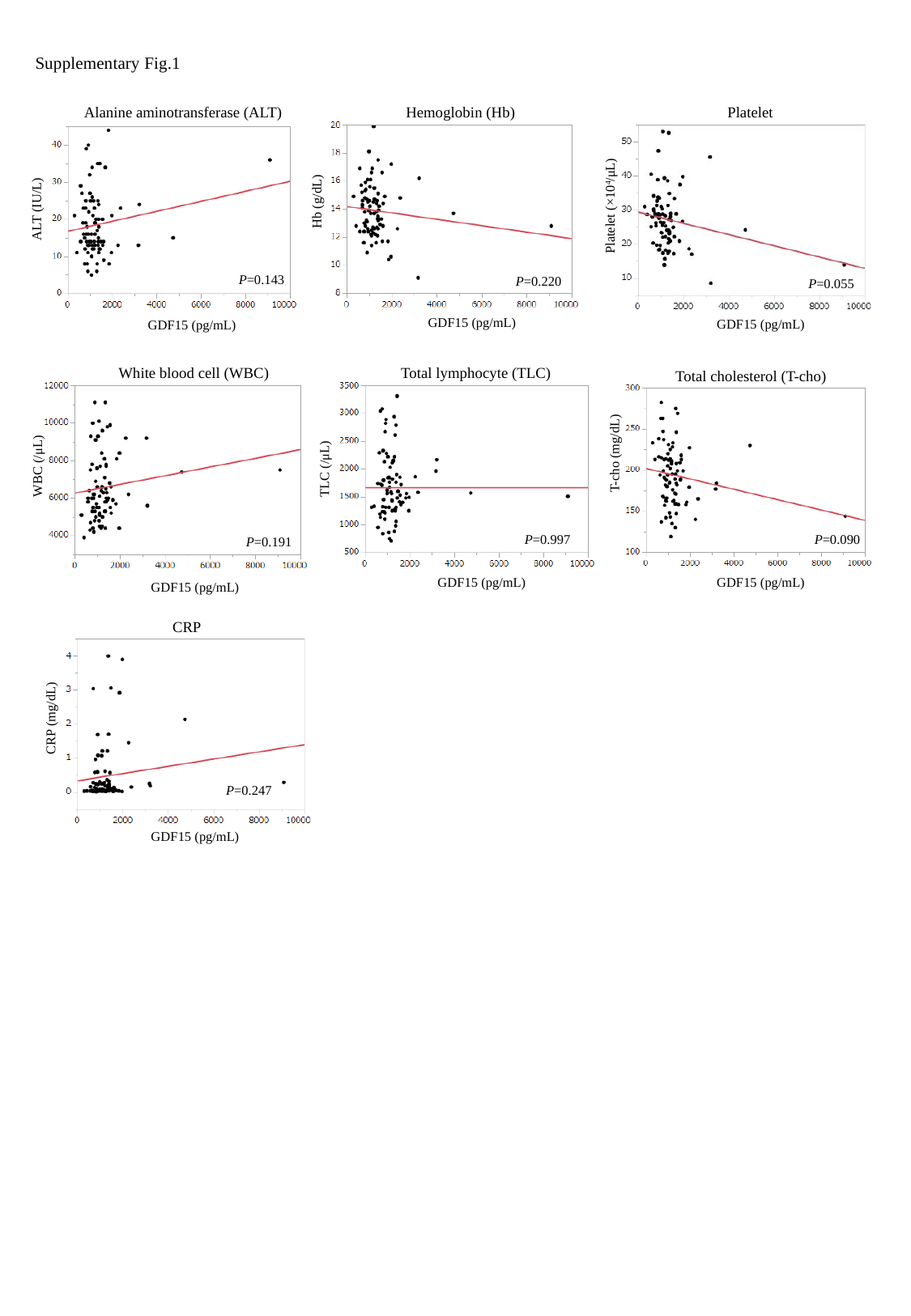

Supplementary Fig.1
Alanine aminotransferase (ALT)
Hemoglobin (Hb)
Platelet
Platelet (×104/μL)
Hb (g/dL)
ALT (IU/L)
P=0.143
P=0.220
P=0.055
GDF15 (pg/mL)
GDF15 (pg/mL)
GDF15 (pg/mL)
White blood cell (WBC)
Total lymphocyte (TLC)
Total cholesterol (T-cho)
T-cho (mg/dL)
WBC (/μL)
TLC (/μL)
P=0.997
P=0.090
P=0.191
GDF15 (pg/mL)
GDF15 (pg/mL)
GDF15 (pg/mL)
CRP
CRP (mg/dL)
P=0.247
GDF15 (pg/mL)
