# Supplementary figures and images for: Correlation of growth differentiation factor 15 level in esophageal cancer with cachectic indicators and postoperative infectious complication
Source: Esophagus. 2025 Sep 29;23(1):230–8. doi: 10.1007/s10388-025-01157-0 (PMC12832575; doi:10.1007/s10388-025-01157-0)

## Slide 1
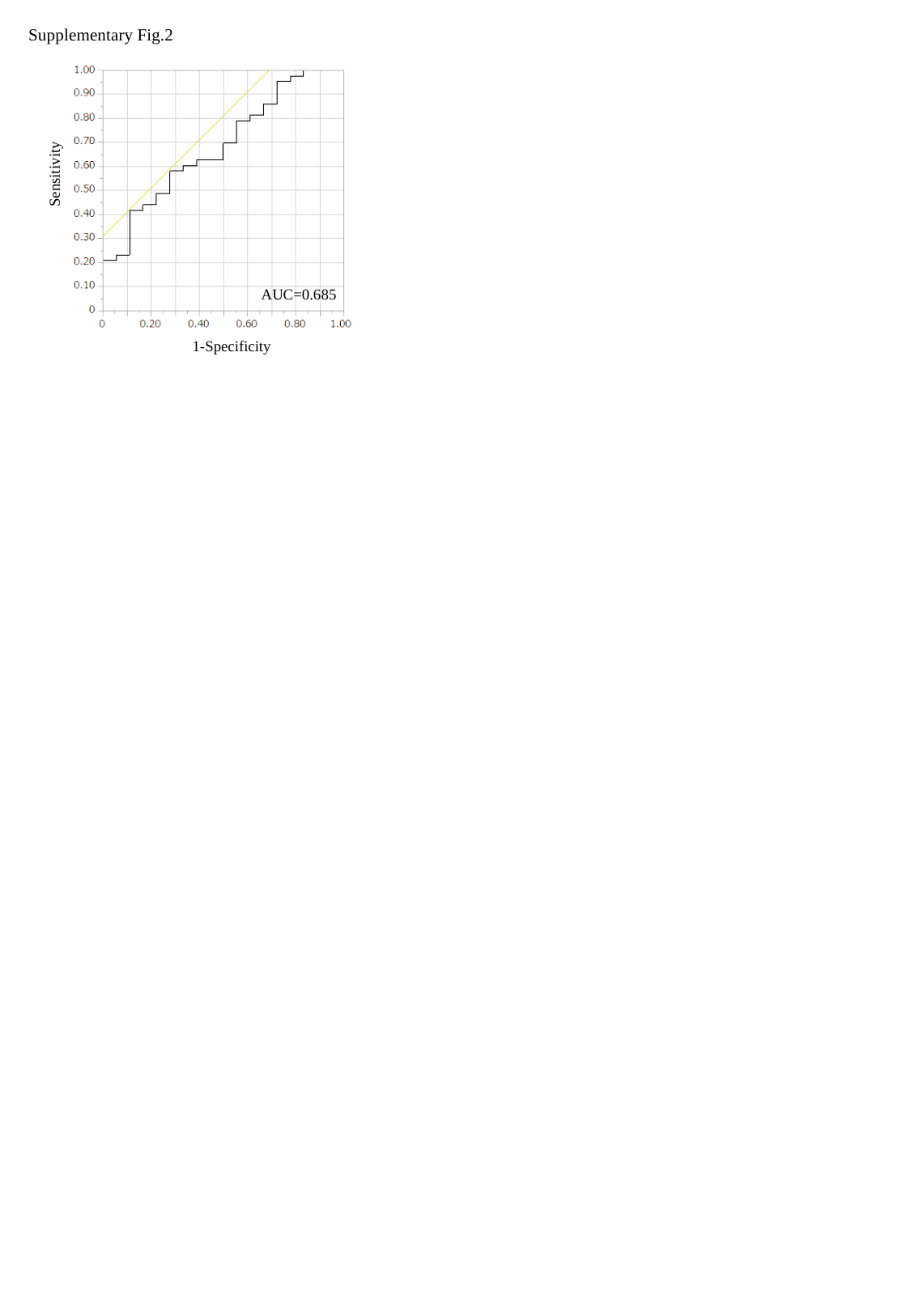

Supplementary Fig.2
Sensitivity
AUC=0.685
1-Specificity

Supplement: Supplementary file 2 — Supplementary file2 Supplementary Fig. 2 Receiver operating characteristic curve for predicting postoperative infectious complication by circulating GDF15. The area under the curve (AUC) of GDF15 is 0.678. The cut-off value is determined to be 940 pg/mL with 42.3% sensitivity and 87.0% specificity. (PPTX 40 KB) [file 10388_2025_1157_MOESM2_ESM.pptx]
